# Trans-scrotal treatment of giant bilateral abdominoscrotal hydroceles in a 7-month-old boy

**DOI:** 10.1016/j.eucr.2023.102469

**Published:** 2023-06-21

**Authors:** Sidney Heersche, Isabelle Vidal, Enrico Brönnimann, Jacques Birraux

**Affiliations:** University Center of Pediatric Surgery of Western Switzerland, Division of Child and Adolescent Surgery, Department of Women, Child and Adolescent, Geneva University Hospitals, Department of Pediatrics, Gynecology and Obstetrics, University of Geneva, Rue Willy-Donzé 6, 1205, Geneva, Switzerland

**Keywords:** Abdominoscrotal hydrocele, Giant hydrocele, Infant, Scrotal approach

## Abstract

Abdominoscrotal hydrocele (ASH) is a rare condition characterized by a large scrotal and abdominal fluid-filled sac. An inguinal surgical approach is generally described in literature.

We report the case of a 7-month-old child who underwent surgical repair of bilateral ASH through bilateral transverse scrotal incisions. The scrotal approach enabled optimal visibility and access to the hydrocele sacs. Separation of the sac from the testicular pedicle was possible with excellent control. Complete excision of the sac was performed. The postoperative course was uneventful. Follow-up after three years shows an excellent result.

We recommend ASH repair through a transverse scrotal incision.

## 1 Introduction

Abdominoscrotal hydroceles (ASH) are large scrotal and retroperitoneal fluid-filled sacs, with an incidence between 0.4 and 3.1%^1^^,^^2^ of all pediatric hydroceles. Bilateral ASH are even more uncommon.

Surgery is performed to avoid discomfort in day-to-day life (i.e. inability to sit, walk, or wear diapers) and to avoid future complications, such as uretero-hydronephrosis, lymphedema, testicular dimorphism, interrupted spermatogenesis, testicular torsion, spontaneous rupture or hemorrhage.^3^

Many surgical approaches are possible for surgical repair, the inguinal incision being the generally preferred approach, although at risk of damage to the elements of the spermatic cord.

We present a bilateral scrotal approach as a better alternative.

## 2 Case presentation

Our patient was a 3-month-old boy initially followed for distal hypospadias, but bilateral hydroceles were found during physical examination. They progressed to giant ASHs in the following months. Parents reported difficulty fitting diapers and discomfort while sitting. The hydroceles were palpated on both sides of the umbilicus, with positive *trans*-illumination and positive springing back ball sign.

**Fig. 1:**
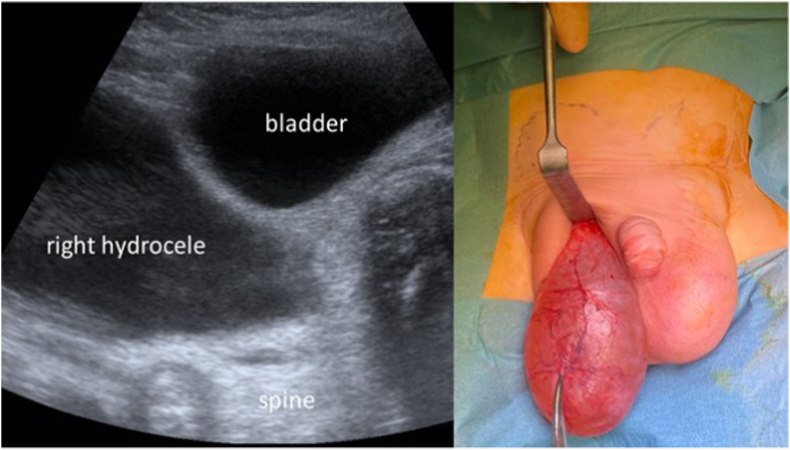
Abdominal component of the right hydrocele (transversal sonographic view), Scrotal component of the hydrocele (intraoperative).

Abdominal ultrasound showed two ASHs, ending at the inferior poles of the kidneys (Fig. 1). Testicles were intra-scrotal and of normal morphology.

**Fig. 2:**
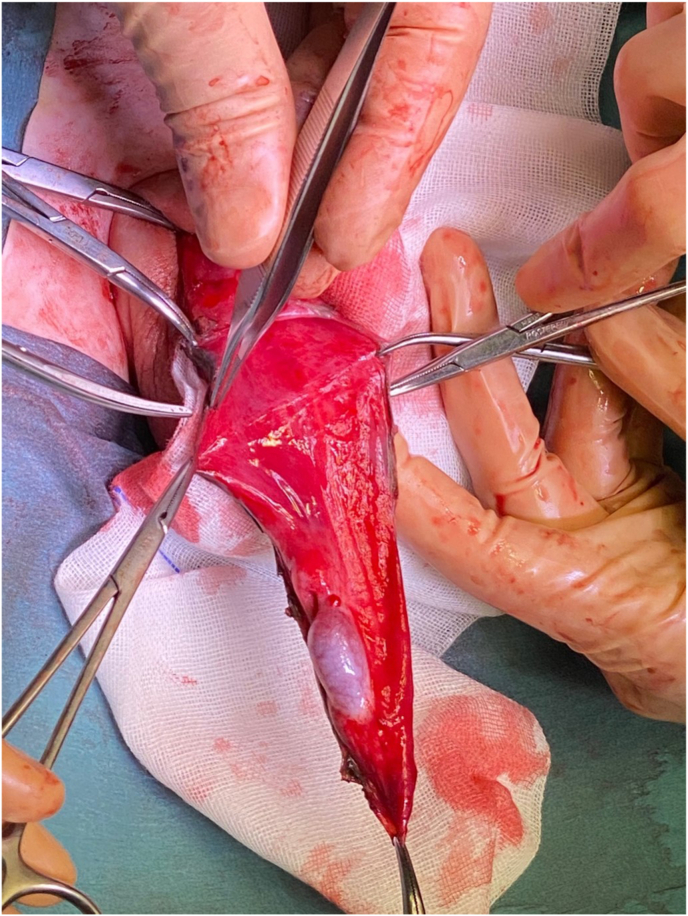
Dissection of the elements of the spermatic cord from the sac.

**Fig. 3:**
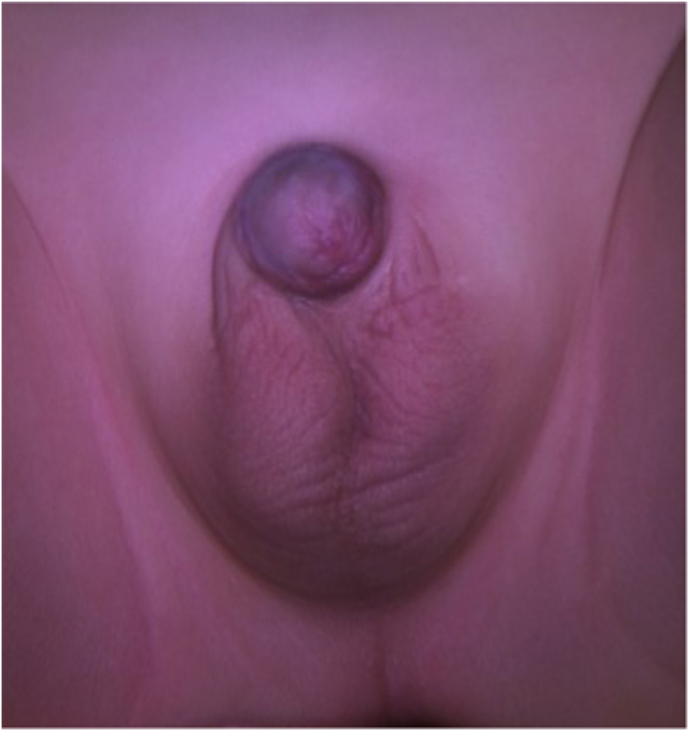
Final result at 3 years post-operation.

Surgical repair was performed at seven months of age. Through a right transverse scrotal incision, the dartos was opened to the tunica vaginalis to create a dissection plane. The scrotal component was completely freed (Fig. 1). Puncture and partial drainage of the sac allowed further upward dissection. The tunica vaginalis was then opened, allowing the dissection of the parietal layer from the spermatic cord (Fig. 2). The abdominal component could then be dissected. The same technique was performed on the left side, with a total of 150 ml drained from the right side and 100 ml from the left. One dose of intravenous amoxicillin/clavulanic acid was administered and was continued orally for 48h. There were no peri-operative complications. The patient was discharged on the second postoperative day. A follow-up 4-month post-operative ultrasound showed no recurrence and adequate testicular morphology. The excellent cosmetic result is shown in Fig. 3, taken 3 years after surgery.

## 3 Discussion

The first mention of ASH was in 1834 by Dupuytren,^4^ who described a “Hydrocèle en bissac”, referencing its hourglass shape. The cause of ASH remains unknown, the most accepted theory assumes that the distension of the tunica vaginalis leads to superior displacement of the hydrocele into the low-pressure abdominal cavity through the inexpansible inguinal canal.^1^

Diagnosis can be made as in our case by clinical exam and ultrasound. Further investigations should be conducted if the diagnosis remains unclear or a mass effect is seen.

In addition to the concern of secondary complications, the cosmetic and mechanical implications of the hydrocele can also be indications for surgery. In our case, the parents had difficulty fitting diapers and remarked discomfort when sitting due to bilateral masses.

Many surgical techniques have been established for the treatment of ASH, with the inguinal approach often being preferred. However, this approach may be challenging because of the suboptimal access to the scrotal component and possible adherences and distortion of the components of the inguinal canal which may threaten the survival of the testicles.^5^ We have chosen the scrotal approach, which provides excellent access to the scrotal part of the hydrocele. We were also pleasantly surprised by the easy dissection of the abdominal components following partial drainage of fluid. Once the hydrocele opened, the structures of the spermatic cord were placed under gentle tension avoiding any distortion and facilitating identification and separation from the tunica vaginalis. Through this approach, bilateral repair in the same setting was feasible. Furthermore, a shorter hospital stay, fewer post-operative complications, and better cosmetic aspect of the scar have been reported compared to the inguinal approach.^5^

Surgical complications of giant ASH repair described in the literature are section of the vas deferens, scrotal hematoma, persistent scrotal swelling, inguinal hernia, and hypoplastic testis.^1^ In their review of 18 patients with 23 cases of ASH, Cozzi et al.^1^ found the scrotal approach to have fewer complications compared to the inguinal approach. We did not encounter any complications. A 4-month post-operative ultrasound showed no recurrence and normal testicular morphology. Long-term cosmetic results were excellent.

## 4 Conclusion

Bilateral abdominoscrotal hydroceles are rare in children. Surgical repair is indicated to prevent secondary complications or in case of mechanical implications. We recommend repair through bilateral transverse scrotal incisions. Its simple access to the sac, bilateral repair in one setting, satisfactory visibility ensuring the safety of the spermatic cord, and excellent cosmetic results make it our approach of choice.

## Consent

Written consent for this case report and use of images was obtained from the child's legal guardian.

## Generative AI and AI assisted technologies in the writing process

No generative AI or AI-assisted technologies were used in the writing process.

## Declaration of competing interest

The authors declare no conflict of interest.

## References

[1] Costantino E., Ganesan G.S., Plaire J.C. (2017). Abdominoscrotal hydrocele in an infant boy. BMJ Case Rep.

[2] Avolio L., Chiari G., Caputo M.A., BragherI R. (2000). Abdominoscrotal hydrocele in childhood: is it really a rare entity?. Urology.

[3] Kamble P.M., Deshpande A.A., Thapar V.B., Das K. (2015). Large abdominoscrotal hydrocele: uncommon surgical entity. Int J Surg Case Rep.

[4] Dupuytren G. (1836).

[5] Kajbafzadeh A.M., Talab S.S., Elmi A. (2010). Modified scrotal approach for correction of abdominoscrotal hydrocele in children: clinical presentation and description of technique. Urology.

